# The extent to which soil hydraulics can explain ecohydrological separation

**DOI:** 10.1038/s41467-022-34215-7

**Published:** 2022-10-30

**Authors:** Catherine E. Finkenbiner, Stephen P. Good, J. Renée Brooks, Scott T. Allen, Salini Sasidharan

**Affiliations:** 1 Department of Biological & Ecological Engineering, Oregon State University, Corvallis, OR USA; 2 Water Resources Graduate Program, Oregon State University, Corvallis, OR USA; 3 Pacific Ecological Systems Division, Center for Public Health and Environmental Assessment, United States Environmental Protection Agency, Corvallis, OR USA; 4 Department of Forest Ecosystems & Society, Oregon State University, Corvallis, OR USA; 5 Department of Natural Resources & Environmental Science, University of Nevada, Reno, NV USA

**Keywords:** Hydrology, Environmental chemistry

## Abstract

Field measurements of hydrologic tracers indicate varying magnitudes of geochemical separation between subsurface pore waters. The potential for conventional soil physics alone to explain isotopic differences between preferential flow and tightly-bound water remains unclear. Here, we explore physical drivers of isotopic separations using 650 different model configurations of soil, climate, and mobile/immobile soil-water domain characteristics, without confounding fractionation or plant uptake effects. We find simulations with coarser soils and less precipitation led to reduced separation between pore spaces and drainage. Amplified separations are found with larger immobile domains and, to a lesser extent, higher mobile-immobile transfer rates. Nonetheless, isotopic separations remained small (<4‰ for δ^2^H) across simulations, indicating that contrasting transport dynamics generate limited geochemical differences. Therefore, conventional soil physics alone are unlikely to explain large ecohydrological separations observed elsewhere, and further efforts aimed at reducing methodological artifacts, refining understanding of fractionation processes, and investigating new physiochemical mechanisms are needed.

## Abstract (web summary)

Soil physics simulations show water isotope ratios can differ among drainage, mobile and immobile storages due to transport processes alone, but effects were smaller than field data implying unrepresented processes underly ecohydrologic separation.

## Introduction

Observations of water from soils, plants, and streamflow are geochemically disparate, as was first shown by Brooks et al.^1^ and further explored in subsequent studies^2–6^. These results conflict with traditional hydrologic assumptions where soil layers are well-mixed water reservoirs wherein new precipitation completely mixes with previously stored water before seeping downward or being absorbed by roots for transpiration. Separations (defined here as contemporaneous isotopic differences among waters stored in different pore spaces within soils or between soil storages and drainage from soils) in tracer values have specifically been reported among water traveling through preferential flow paths, water residing and tightly held within a soil matrix, and water discharged as drainage across various soil types^2,7–11^ and climates^1,3,12–15^. Stable water isotopes ratios of hydrogen (^2^H/^1^H) and oxygen (^18^O/^16^O), hereafter communicated as δ^2^H and δ^18^O, in precipitation, soil water, and plant root extracts have been utilized as tracers to investigate transport^5^ and so-called ecohydrological separations^2–4,6^. Separate from those observational studies of isotopic differences between pore spaces, new theoretical frameworks have shown how flow through systems composed of contrasting conductivities will support transport dynamics that lead to separations between storages and fluxes^16–18^. We expand on these frameworks previously applied to individual sites and case studies to explore, more generally, how soil physical properties across a range of soils and climates may contribute to ecohydrological separation caused by transport heterogeneity in soils.

One of the difficulties of modeling observed separations is that traditional hydrologic modeling approaches fail to account for heterogeneous mixing and transport processes by assuming incoming precipitation enters the soil matrix and mixes fully with pre-existing soil water. Several numerical modeling studies have attempted to represent separations arising in soils^17,19–24^. These studies have advanced understanding of subsurface mixing by quantifying flow heterogeneities in the unsaturated zone and the spatiotemporal variability of water and solute transport. Sprenger et al.^23^ investigated separations between soil water pore spaces using a one-dimensional flow model at three long-term northern latitude research sites and found that modeling with two pore domains improved the representation of soil water isotope dynamics when compared to field measurements. We expand on this work by utilizing a fully mechanistic modeling approach to examine how separation phenomena may arise across a wide range of soil hydraulic parameters or climate conditions to quantify which environmental conditions do (or do not) contribute to the separation phenomena reported in other studies^1,16,25^.

We configured 650 isotope-enabled soil physics models (HYDRUS-1D^26,27^) to evaluate soil water transport and mixing across various soil types and simulated precipitation from different climates. This approach explicitly allowed for the heterogeneous mixing of solutes at depth via a dual-porosity method. We explored to what extent ecohydrological separations (or lack thereof) could be explained by soil physical properties alone. Specifically, our research objective was to test how heterogeneous flow through porous media, without confounding evaporative or instrumental effects^14^, could yield differences in the isotopic composition between water in preferential flow paths (i.e., mobile pore space), water tightly-bound between soil pores (i.e., immobile pore space), and water discharged from the soil column as drainage. First, we configured a model representative of one soil type, roughly similar to soils at H.J. Andrews studied by Brooks et al.^1^, and tested if unique separations arose using a single or dual-porosity model design. The single porosity model was compared to dual-porosity models with smaller and larger immobile domains (relative to the bulk volumetric water content) and slower and faster mass transfer rates between immobile and mobile domains. Then, we varied the soil hydraulic properties of saturated water content and saturated hydraulic conductivity, as well as climate properties of total precipitation amounts and the correlation between precipitation isotopic composition and amount. This work extends beyond previous studies by identifying how interactions between climate and pore-space heterogeneity within a soil profile drive incomplete mixing in soils across a wide range of conditions. These results help frame how we understand and represent solute transport and mixing in the subsurface.

## Results

### Understanding dual-porosity isotope separation in soils

**Fig. 1:**
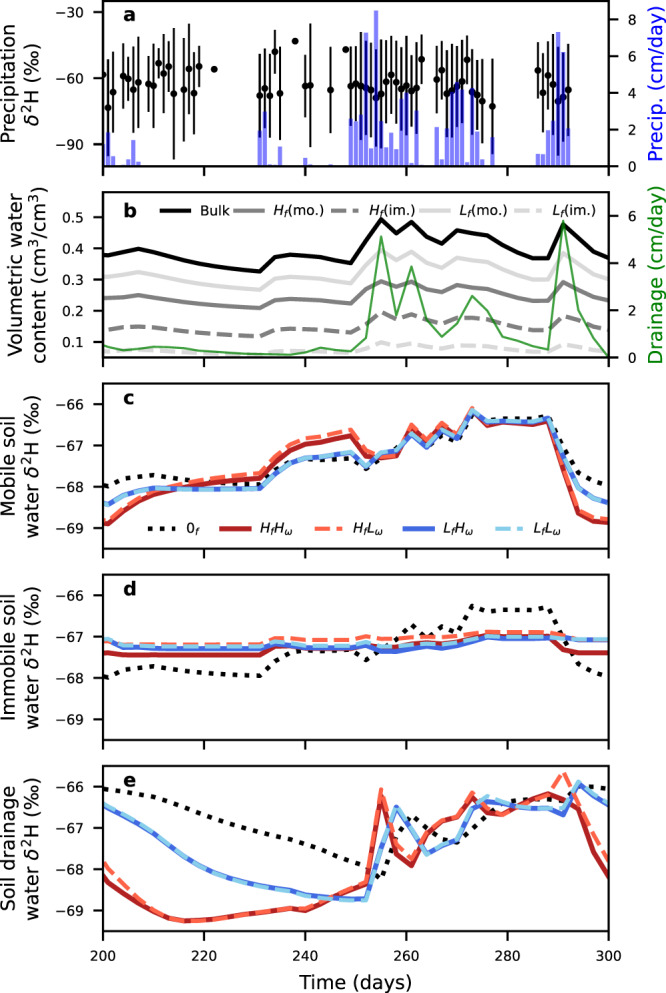
Simulated precipitation, soil pore space waters, and drainage isotope ratios. **a** Averaged downscaled flux-weighted precipitation δ^2^H isotope value with error bars denoting ±1 standard deviation from the ten input simulations as well as the daily input precipitation amount as blue bars. **b** Averaged time series of the ten simulated volumetric water contents of a single porosity soil (Bulk, solid black line), the mobile and immobile volumetric water contents for a soil with high immobile fractions (*H*_*f*_(mo.) and *H*_*f*_(im.) respectively, dark gray), and the mobile and immobile volumetric water contents for a soil with low immobile fractions (*L*_*f*_(mo.) and *L*_*f*_(im.) respectively, light gray), as well as the drainage from the column (thin green line). δ^2^H isotope ratios of the **c** mobile soil water domain, **d** immobile soil water domain, and **e** column drainage for the soils simulated with a single pore domain model (0_*f*,_ black), high immobile fraction with high and low transfer rates (*H*_*f*_*H*_*ω*_, red; *H*_*f*_*L*_*ω*_, orange) and low immobile fractions with high and low transfer rates (*L*_*f*_*H*_*ω*_, cyan; *L*_*f*_*L*_*ω*_, blue). The input precipitation time series was repeated three times in series, with the final 100 days (days 200–300) displayed. Note: line styles are the same for **c**–**e** and the line for *L*_*f*_*H*_*ω*_ is behind *L*_*f*_*L*_*ω*_ in **d** and **e**.

Isotopic separations were evaluated across simulations of different hypothetical conceptualizations of water transport for a single soil type using a single porosity model and a dual-porosity model with high (40%) and low (20%) immobile fractions and high (0.75) and low (0.25) mass-transfer rates between the mobile and immobile domains (Fig. 1). Across the range of fractions of pore space in the immobile domain and mass transfer coefficients between the mobile and immobile domains, soils with immobile fractions exhibited distinct transport processes that were not apparent with a single porosity soil configuration (Fig. 1c–e). Given that these models were driven with the same inputs and all other model parameters were held constant, the temporal patterns which arose were attributable to differences in simulated pore heterogeneities. Immobile soil water had limited δ^2^H variability regardless of precipitation driven changes in bulk volumetric water content or drainage rates (Fig. 1d), which were controlled by dynamics in the mobile domain. This contrasts with mobile-soil and drainage waters, wherein δ^2^H varied in time considerably during wetter and drier simulation periods. When parameterizations included large fractions of immobile soil water, vertical water transport was reduced, and mobile water fractions decreased; however, this manifested in more rapid breakthrough curves that preceded the low immobile fraction breakthrough curves (see days 250-260, Fig. 1e). These differences in tracer breakthrough dynamics occurred because vertical movement within the soil column and mixing of precipitation inputs was limited to the mobile domain, and thus smaller immobile pore volumes implied more mixing within the column. Varying mobile-immobile transfer coefficients did not influence the breakthrough curve dynamics. While the separations between pore spaces were apparent in our simulations, overall differences in δ^2^H from each domain and model configuration were relatively small (all δ^2^H ratios ranged from −70.5‰ to −65.3‰).

**Fig. 2:**
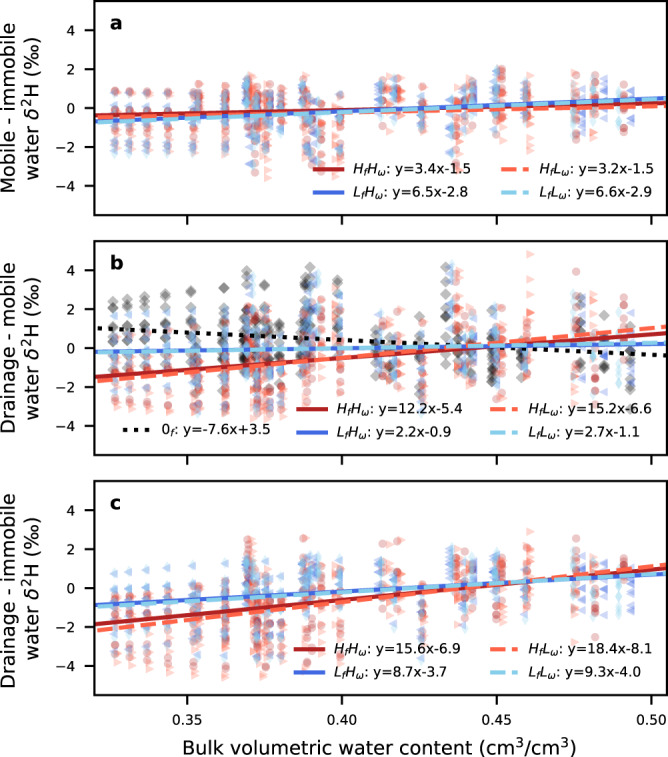
Isotope separation during different soil moisture conditions. The instantaneous isotopic separation for 10 simulations expressed as the δ^2^H difference in **a** isotopic composition of the mobile and immobile pore space water at different volumetric water contents for models with high and low immobile fractions (*H*_*f*_ and *L*_*f*_) and transfer rates (*H*_ω_ and *L*_ω_). The separation in the isotopic composition of the drainage from the column and **b** mobile soil water and **c** immobile water at different water contents.

Consistently across soil configurations, as the bulk volumetric water content and drainage increased, the separation between pore spaces shifted so the drainage water more closely reflected the mobile and immobile water (i.e., the difference between drainage and mobile or immobile water $\cong$ 0‰; Fig. 2b, c) until at a relatively high bulk volumetric water content when the differences reversed (i.e. drainage water was isotopically more positive than mobile water). During drier periods, stronger absolute separations were observed between soil pore spaces and the drainage water (Fig. 2b, c). This difference was likely a function of the incomplete mixing of the soil column with new incoming precipitation and the time-lag between the drained water and water in the mobile domain. As bulk volumetric water content and drainage increased, models with a higher immobile fraction had the largest change in isotopic separation (i.e. more positive slopes in Fig. 2b, c; Supplemental Tables S1 and S3), with all dual-porosity models demonstrating this relationship (most slopes had significant *p*-values <0.05; Supplemental Table S1) and models with higher immobile fractions had stronger changes in separations (i.e. steeper slopes). Interestingly, the separation between the soil and drainage water simulated by a single porosity model had a contrasting relationship (i.e. negative slope), with separations decreasing as bulk volumetric water content increased; this was distinctly different from all other models (Fig. 2b). Within a specific model configuration, variability in the strength of relationships between isotopic separation and hydrologic state arises as a function of stochasticity within the ensemble of simulated isotope precipitation time series which served as input for each of the simulations. Accordingly, changes in the incoming precipitation would likely change the separation dynamics; however, differences among modeled separations should still be apparent across soils with smaller or larger immobile fractions. Supplemental Tables S1–S3 summarize the linear regression, p-value, Pearson correlation coefficient, and Spearman correlation coefficient calculated between isotopic separations and bulk volumetric water content, daily accumulated precipitation, and drainage. Consistently, we find that periods with drier soil water contents will have larger absolute separations between drainage waters and mobile or immobile waters (Fig. 2b, c) and this relationship is amplified for soils with larger immobile fractions.

### Dual-porosity isotope separation across soils and climates

**Fig. 3:**
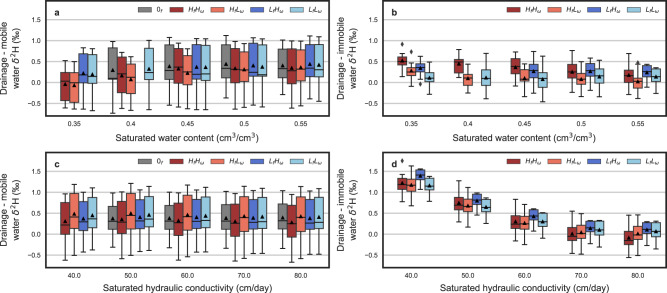
The influence of soil hydraulic parameters on isotope separation. The effect across model simulations of varying soil saturated water content on the average δ^2^H difference between drainage and **a** mobile or **b** immobile soil water as well as the influence of varying soil saturated hydraulic conductivity on the average δ^2^H difference between drainage and **c** mobile or **d** immobile soil water. Differences are show as boxplots for models with high and low immobile fractions (*H*_*f*_ and *L*_*f*_) and transfer rates (*H*_ω_ and *L*_ω_) as well as for a single porosity column (0_*f*_). Each boxplot represents flux and volumetrically weighted averaged differences, with the box spanning upper and lower quartiles, whiskers extending 1.5 times the interquartile range, diamonds as outliers, and black triangles indicating the mean calculated from 10 simulations. Missing boxplots at 0.35 cm^3^/cm^3^ in **a** and **b**, 0.40 cm^3^/cm^3^ in **a** and **b**, and 40 cm/day in **c** and **d** indicate where HYDRUS-1D model configurations failed to converge.

Moving beyond a representation of one soil type, we explored a wide range of soil and climate characteristics with varying immobile fractions and transfer rates. Changing a soil’s total saturated water content and saturated hydraulic conductivity influenced the transport and mixing of soil waters, thereby producing different degrees of separation between mobile and immobile pore spaces and drainage waters (Fig. 3a–d). Separations between the mobile and immobile pore spaces were more pronounced in simulations with lower saturated water content and hydraulic conductivity (Supplemental Fig. S1). Lowering the saturated water content produced the largest separations in soils with high immobile pore fractions and mobile-immobile transfer rates (Supplemental Fig. S1a), accordingly more tightly bound water pore spaces and low saturated water contents drive these larger separations. Furthermore, soils with more tightly bound water pore spaces and low saturated water content demonstrated decreases in the degree of separation between drainage and mobile water, and this decrease was not observed for models with less tightly bound pore spaces (Fig. 3a). Lowering the saturated water content and increasing the strength of transfer between the mobile and immobile domains increased the separation between drainage and immobile water (Fig. 3b). Presumably, this is because the stochastically similar rainfall amounts between models must now transit through less pore space (or transit more slowly), funneling more flow to the mobile domain. Changing the saturated hydraulic conductivity influenced the separation between pore spaces as well, wherein soils with lower values exhibited larger separations between drainage and tightly bound water (Fig. 3d). However, varying the saturated hydraulic conductivity of the soil had a minimal effect on the difference between drainage and mobile water (Fig. 3c). Based on 2-sample *t*-tests, none of the dual-porosity models were statistically different compared to the single porosity model (p-values > 0.5; Fig. 3). It is worth mentioning that all simulated differences in Fig. 3a–c varied by only ~0.5‰ and this difference would be difficult to observe from field observations given typical instrumental accuracy. Regardless of the small separations observed, our results indicate that in soils with low saturated volumetric water content and hydraulic conductivity, the modeling of subsurface drainage and transport processes is most sensitive to representations of mobile and immobile pore spaces.

**Fig. 4:**
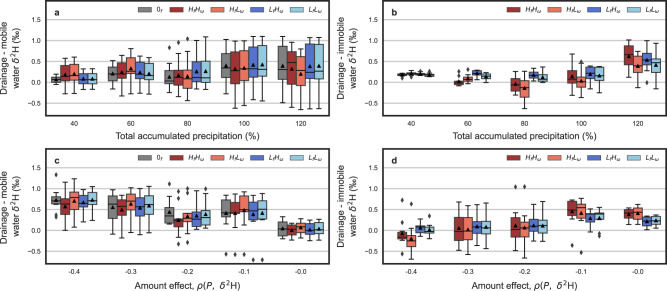
The influence of climate parameters on isotope separation. The effect across model simulations of varying the total input precipitation on the average δ^2^H difference between drainage and **a** mobile or **b** immobile soil water as well as the influence of varying the amount effect’s negative correlation represented by the Pearson correlation coefficient, *ρ*, between precipitation amount, *P*, and its δ^2^H composition on the average δ^2^H difference between drainage and **c** mobile or **d** immobile soil water. Precipitation is shown as a percent of the baseline amount (=101.5 cm). Differences are show as boxplots for models with high and low immobile fractions (*H*_*f*_ and *L*_*f*_) and transfer rates (*H*_ω_ and *L*_ω_) as well as for a single porosity column (0_*f*_). Each boxplot represents flux and volumetrically weighted averaged differences, with the box spanning upper and lower quartiles, whiskers extending 1.5 times the interquartile range, diamonds as outliers, and black triangles indicating the mean calculated from 10 simulations.

Next, we showed that precipitation input amounts can drive the degree of separation between mobile and immobile pore spaces and drainage waters (Fig. 4). We both increased and decreased the total input precipitation in simulations, since precipitation drove increases in soil water content and drainage in the previous H.J. Andrews soil results. Soils receiving less precipitation yielded lower variances in the separations between mobile and immobile pore spaces and drainage waters (Fig. 4a, b and Supplemental Fig. S2a). For these simulations, the soil had a high saturated hydraulic conductivity (65.64 cm/day) and therefore smaller precipitation events were less likely to saturate the soil column. This influenced the rate of mass transfer between mobile and immobile regions, which was driven by a pressure head gradient, thus decreasing the separations simulated at lower precipitation rates.

Given that precipitation amount controls both separation and stable isotope ratios^4,17,28,29^, we posit that the interaction between these two relationships could be a significant factor contributing to the ecohydrologic separation phenomena. We tested if precipitation driven separations were disparate between single and heterogeneous porosity landscapes. We show that varying the strength of the amount effect on precipitation isotopic values, which describes the often-negative correlation (*ρ*) between precipitation amount and its isotopic ratio, influences separations between pore spaces (Fig. 4). A large amount effect (more negative *ρ*) means higher intensity rainfall will have much lower δ^2^H ratios than low intensity rainfall. The value of *ρ* can vary depending on the source of precipitation (e.g., oceanic versus continental precipitation), latitude, seasonal temperatures, or precipitation patterns^30,31^. Observed daily values of *ρ* can range from +0.24 to −0.47^32^, and an amount effect of zero removes any correlation between precipitation intensity and its isotopic composition. The most negative precipitation events were simulated when the amount effect was strong (*ρ* = −0.4) and the input precipitation amount was large. Consequently, these simulations resulted in the largest separations between mobile and immobile soil water (Supplemental Fig. S2b) and between drainage and mobile soil water (Fig. 4c). The larger separations can be attributed to the large, negative precipitation events entering the mobile soil domain later in the time series when precipitation intensity was the largest. These large, negative precipitation events also shifted the degree of separation between drainage waters and immobile pore spaces (Fig. 4d). Thus, the separations between pore spaces and fluxes attributable to the soil transport processes examined here are likely to be greater in regions with large storm events and large amount effects (i.e., rain-out effects), seasonal precipitation which transitions from snow to rain, or intense temperature changes between seasons.

## Discussion

Many research studies have studied the effects of ecohydrologic separations^1–4,6,15,21–23^ and suggest different pore spaces supply plant transpiration (immobile) and groundwater recharge and streamflow (mobile)^1,25^. Here, we demonstrate how soil properties such as a low saturated volumetric water content and high immobile pore fraction can drive separations between pore spaces supplying plants and other water pools (e.g., streamflow). Our simulations based on soil physics alone showed the largest separations between drainage and mobile or immobile water were ~4‰ in δ^2^H (Fig. 2a) and, when averaged over time, were ~1−2‰ in δ^2^H (Figs. 3a–d and 4a–d). We acknowledge that only a few soil physical properties were investigated here, and other factors not simulated in this study could also drive separations. Literature has cited varying degrees of separation between soils and streams, often far exceeding 4‰^1,33^ (e.g., Brooks et al.^1^ reported differences of ~ 10‰). However, recent literature has shown that these separations are likely overestimated^7,12^ (also refer to Allen and Kirchner^34^). Moreover, our simulated values cannot be directly compared to previous studies since streamflow is primarily composed of temporally integrated groundwater at any given time, and there are much longer time lags involved in streamflow generation^16,33^. We found the range of simulated differences was small (<4‰), yet our results did show that soils represented with heterogeneous porosities are likely to produce larger separations between soi1 and drainage water, and thus such soils are more likely to contribute to previously described isotopic differences between soil water and streamflow.

The implications of varying the size and influence of mobile-immobile pore fractions and transfer coefficients for tracer transport and mixing have not been explicitly investigated in great detail. Prior work by Hu et al.^24^ used the simplifying assumption that half of the total storage was mobile water and no mass transfer occurred between regions, nevertheless they found improved transit time estimates when compared to a complete mixing model. Here, we clarify how increases in the fraction of immobile pore space resulted in a decrease in soil hydrologic connectivity and an increase in the amount of incoming precipitation likely to bypass the matrix. The largest absolute separations between drainage and mobile or immobile water were simulated with large fraction of immobile pore space (Fig. 2b, c). Lowering mobile-immobile transfer rates decreases the exchange between the matrix and preferential flow, further limiting mixing and transfer between domains especially under dry soil conditions. A low mobile-immobile transfer rate often increased the separation between drainage and immobile pore waters (Fig. 3b, d), though research on these rates remains limited. Lab based column^2^ and field scale experiments^35^ suggest that exchange is needed to sustain macropore flow in soils and is influenced by precipitation intensity and antecedent moisture levels. Based on our findings and previous work, we hypothesize that soil representations accounting for both mobile-immobile pore fractions and transfer coefficients will better characterize transport and mixing, as well as prescribing that some subsurface water resides longer (e.g., an increase in immobile pore spaces will increase the time water travels through the matrix), while other configurations will result in more water transported through preferential flow paths.

While specifically excluded here, other factors could drive the larger separations quoted in previous studies. These include accounting for evaporation and the corresponding isotopic fractionation mechanisms^36^ or root water uptake from the mobile or immobile domains^9^, as well as interacting effects of seasonality^37^. It also should be noted that plant and soil water extraction techniques can introduce biases and uncertainties in stable water isotope analyses^7,12,34,38,39^. Many of the simulated differences here are 1−2‰, which is near the precision of many laser-based systems when measuring water samples for δ^2^H. Consequently, the simulated differences between domains found here would be challenging to detected in nature. Future research should build on this analysis through systematic testing of the potential separation magnitudes that can result from other mechanisms (e.g., evaporation, root water uptake, rhizosphere interactions) to quantify the impact of any hypothetical explanation, although hydrologic model representations of those other processes may lag representations of soil water transport and mixing.

In this study, we comprehensively evaluated water transport in the subsurface under different soil porosity heterogeneities across a range of fine to coarse soils and wet, dry, and seasonally varying climate conditions. The separations arising from soil physical properties or climate effects alone are expected to be relatively small. Regardless, we have demonstrated that separations can occur with heterogeneous mixing in soils alone and how these separations are expected to vary with local ecohydrologic characteristics. Thus, any models aiming to realistically represent transport processes, especially those characterized through tracer observations, must represent the heterogeneity of soil pores within a soil column and the exchanges among them. This analysis demonstrated that the coexistence of finer and coarser pores within a single soil profile can manifest complex tracer phenomena, albeit of relatively limited magnitude. Therefore, we conclude that conventional soil physics alone (including dual porosity conceptualizations) are unlikely to drive the large ecohydrological separations reported elsewhere, which clarifies the need for attention to be directed at understanding methodological artifacts, fractionation and related physiochemical processes, and approaches to realistically model those processes so that they can be comprehensively evaluated via simulation and virtual experimentation.

## Methods

### Numerical simulation of soil physics

The one-dimensional HYDRUS-1D dual-porosity numerical model^26^ with modifications for stable water isotope transport^27^ was configured to represent 650 different porosity heterogenies across soils with different hydraulic properties and climate conditions. HYDRUS^26^ is one of the most widely used numerical models for simulating the movement of water, heat, and solutes in various soil conditions and has been used in other isotope studies in a single porosity configuration^14,21,23,27,34,40^. We first configured a dual-porosity HYDRUS-1D model with parameters based on observed soil hydraulic properties from Watershed 10 at H.J. Andrews Experimental Forest, Oregon, USA^1^. Simulations of bulk volumetric water content were compared against observed values from 14 September 2006 to 23 December 2006. These 100 days were selected for our analysis because precipitation events during this period were sampled for their isotopic composition in 5 mm increments, making this a unique dataset on which to test our hypothesis since many other sampling approaches integrate precipitation sampling over a week or longer time scales. The observed precipitation samples were used within a statistical downscaling method^32^ to simulate realizations of possible precipitation inputs corresponding with observed precipitation amounts. The model represented the top 100 cm of the soil profile, no evaporative effects were considered from the surface, and no root water uptake was simulated within the column. All pore water heterogeneities were driven by model parameterization of the soil’s physical properties. Refer to the Supplemental Information for further details on model configuration, parameterization, initial conditions, and the simulated input precipitation datasets.

The modeled mobile soil water domain represented regions of the soil matrix such as preferential flow paths or large pore-spaces, and the modeled immobile soil water domain represented tightly bound water held at water potentials below what can drain by gravity. Water was transported vertically only in the mobile region and water movement into or out of the immobile region was controlled by the pressure head gradient and a mass transfer coefficient^26^. We investigated five model configurations characterizing different heterogeneous mixing and transport by altering the immobile fraction, *f*, and the mobile-immobile transfer rate, *ω*. These configurations are: (1) 0 _*f*_, a model representing a single-pore domain with zero immobile fraction, (2) *H*_*f*_ *H*_*ω*_, a model with a high *f* representing 40% of the total saturated water content and high *ω* equaling 0.75, (3) *L*_*f*_ *H*_*ω*_, a model with a low *f* representing 20% of the total saturated water content and high *ω* equaling 0.75, (4) *H*_*f*_ *L*_*ω*_, a model with a high *f* representing 40% of the total saturated water content and low *ω* equaling 0.25, and (5) *L*_*f*_ *L*_*ω*_, a model with a low *f* representing 20% of the total saturated water content and low *ω* equaling 0.25. These five model configurations were tested across a range of different precipitation inputs and soil hydraulic properties, which were varied in relation to the original soil column configured from observed datasets from Watershed 10, H.J. Andrews Experimental Forest.

### Simulated soil and precipitation characteristics

In total, we present 650 simulations of HYDRUS-1D with varying immobile pore fractions and mobile-immobile transfer rates across different values of saturated water content, saturated hydraulic conductivity, total accumulated precipitation, and correlation between precipitation amount and its isotopic composition (e.g. amount effects^41^). When varying soil hydraulic parameters across model configurations, all other parameters were held constant and saturated water content and hydraulic conductivity were altered (see table S4 for parameter ranges). We increased or decreased the total accumulated precipitation by multiplying each precipitation event in the input time series by a specific percentage. New precipitation isotope ratios were generated (refer to the Supplemental Information) with stronger or weaker negative correlations between precipitation depth and its isotopic ratio. All model simulations had relatively low mass balance errors (average relative error = 0.09%) and solute balance errors (average relative error = 0.001‰) calculated by HYDRUS-1D during the numerical computations^26^.

This study represented 100 days in a Pacific Northwest winter wet season^1,32^ and not a full year with dry periods, large evaporative effects, or high transpiration rates. For similar forests during this period, ecosystem evapotranspiration is a minor component of hydrologic dynamics^42^. The 100-day time series was repeated three times to remove the effects of the initial condition of the soil’s stable water isotope signature, and the final 100 days (days 200-300) were analyzed in the presented results. We chose this approach to reduce the confounding effects of evaporation and we were constrained by the computation limitations imposed by running HYDRUS-1D with many configurations. The observation dataset from Brooks et al.^1^ was used to estimate our modeled soil properties (refer to the Supplemental Information), however our objective was not to exactly simulate the observed water isotope datasets. The simulated stable water isotopes in precipitation are not equal to the actual precipitation during the period, and we did not know the initial soil water isotopic value. Our focus was to test the dual-porosity approach across 650 representations of transport dynamics across soils and climates.

## Supplementary information

Supplementary Information

## Data Availability

The HYDRUS model output data generated in this study have been deposited in the CUAHSI HydroShare Database^43^. Meteorological data for H.J. Andrews from 1975 to present is available from the Environmental Data Initiative^44^.

## Code availability

The software used in this study is freely available at https://www.pc-progress.com/. Supplementary Table S4 contains the ranges of parameters used in this study with further model configuration information available from the corresponding author with reasonable request.

## References

[1] Brooks JR, Barnard HR, Coulombe R, McDonnell JJ (2010). Ecohydrologic separation of water between trees and streams in a Mediterranean climate. Nat. Geosci..

[2] Radolinski, J., Pangle, L., Klaus, J. & Stewart, R. D. Testing the ‘two water worlds’ hypothesis under variable preferential flow conditions. *Hydrol. Process*. **35**, e14252 (2021).

[3] Sprenger M, Llorens P, Cayuela C, Gallart F, Latron J (2019). Mechanisms of consistently disjunct soil water pools over (pore) space and time. Hydrol. Earth Syst. Sci..

[4] McDonnell JJ (2014). The two water worlds hypothesis: ecohydrological separation of water between streams and trees?. Wiley Interdiscip. Rev. Water.

[5] Stumpp C, Nützmann G, Maciejewski S, Maloszewski P (2009). A comparative modeling study of a dual tracer experiment in a large lysimeter under atmospheric conditions. J. Hydrol..

[6] Sprenger M, Leistert H, Gimbel K, Weiler M (2016). Illuminating hydrological processes at the soil-vegetation-atmosphere interface with water stable isotopes. Rev. Geophys..

[7] Barbeta A (2020). An explanation for the isotopic offset between soil and stem water in a temperate tree species. N. Phytol..

[8] Berry ZC (2018). The two water worlds hypothesis: Addressing multiple working hypotheses and proposing a way forward. Ecohydrology.

[9] Dubbert M, Caldeira MC, Dubbert D, Werner C (2019). A pool‐weighted perspective on the two‐water‐worlds hypothesis. N. Phytol..

[10] MAŁOSZEWSKI P (2006). Modelling of water flow through typical Bavarian soils: 2. Environmental deuterium transport. Hydrol. Sci. J..

[11] Vargas AI, Schaffer B, Yuhong L, Sternberg LDSL (2017). Testing plant use of mobile vs immobile soil water sources using stable isotope experiments. N. Phytol..

[12] Chen Y (2020). Stem water cryogenic extraction biases estimation in deuterium isotope composition of plant source water. Proc. Natl Acad. Sci. USA.

[13] Mueller MH (2014). Tracking water pathways in steep hillslopes by δ18O depth profiles of soil water. J. Hydrol..

[14] Stumpp C, Hendry MJ (2012). Spatial and temporal dynamics of water flow and solute transport in a heterogeneous glacial till: The application of high-resolution profiles of δ18O and δ2H in pore waters. J. Hydrol..

[15] Sprenger, M. & Allen, S. T. What ecohydrologic separation is and where we can go with it. *Water Resour. Res*. **56**, e2020WR027238 (2020).

[16] Berghuijs WR, Kirchner JW (2017). The relationship between contrasting ages of groundwater and streamflow. Geophys. Res. Lett..

[17] Cain MR, Ward AS, Hrachowitz M (2019). Ecohydrologic separation alters interpreted hydrologic stores and fluxes in a headwater mountain catchment. Hydrol. Process..

[18] Smith AA, Tetzlaff D, Soulsby C (2020). Using storage selection functions to assess mixing patterns and water ages of soil water, evaporation and transpiration. Adv. Water Resour..

[19] Knighton, J. et al. Using isotopes to incorporate tree water storage and mixing dynamics into a distributed ecohydrologic modelling framework. *Ecohydrology* **13**, e2201 (2020).

[20] Stumpp C, Maloszewski P (2010). Quantification of preferential flow and flow heterogeneities in an unsaturated soil planted with different crops using the environmental isotope δ18O. J. Hydrol..

[21] Sprenger M, Volkmann THM, Blume T, Weiler M (2015). Estimating flow and transport parameters in the unsaturated zone with pore water stable isotopes. Hydrol. Earth Syst. Sci..

[22] Sprenger M, Seeger S, Blume T, Weiler M (2016). Travel times in the vadose zone: Variability in space and time. Water Resour. Res..

[23] Sprenger M (2018). Measuring and modeling stable isotopes of mobile and bulk soil water. Vadose Zone J..

[24] Hu H, Dominguez F, Kumar P, McDonnell J, Gochis D (2018). A numerical water tracer model for understanding event-scale hydrometeorological phenomena. J. Hydrometeorol..

[25] Evaristo J, Jasechko S, McDonnell JJ (2015). Global separation of plant transpiration from groundwater and streamflow. Nature.

[26] Šimünek, J., Šejna, M., Saito, H., Sakai, M. & VanGenuchten, M. *The HYDRUS-1D software package for simulating the one-dimensional movement of water, heat, and multiple solutes in variably-saturated media – version 4.17*. (2013).

[27] Stumpp C, Stichler W, Kandolf M, Šimůnek J (2012). Effects of land cover and fertilization method on water flow and solute transport in five lysimeters: a long-term study using stable water isotopes. Vadose Zone J..

[28] Kirchner JW (2016). Aggregation in environmental systems-Part 1: Seasonal tracer cycles quantify young water fractions, but not mean transit times, in spatially heterogeneous catchments. Hydrol. Earth Syst. Sci.

[29] Sprenger M, Tetzlaff D, Soulsby C (2017). Soil water stable isotopes reveal evaporation dynamics at the soil–plant–atmosphere interface of the critical zone. Hydrol. Earth Syst. Sci..

[30] Gat JR (1996). Oxygen and hydrogen isotopes in the hydrologic cycle. Annu. Rev. Earth Planet. Sci..

[31] Good SP, Noone D, Kurita N, Benetti M, Bowen GJ (2015). D/H isotope ratios in the global hydrologic cycle. Geophys. Res. Lett..

[32] Finkenbiner CE, Good SP, Allen ST, Fiorella RP, Bowen GJ (2021). A statistical method for generating temporally downscaled geochemical tracers in precipitation. J. Hydrometeorol..

[33] Rodriguez NB, McGuire KJ, Klaus J (2018). Time‐varying storage–water age relationships in a catchment with a mediterranean climate. Water Resour. Res..

[34] Allen, S. T. & Kirchner, J. W. Potential effects of cryogenic extraction biases on plant water source partitioning inferred from xylem‐water isotope ratios. *Hydrol. Process*. **36**, e14483 (2022).

[35] Klaus J, Zehe E, Elsner M, Külls C, McDonnell JJ (2013). Macropore flow of old water revisited: experimental insights from a tile-drained hillslope. Hydrol. Earth Syst. Sci..

[36] Zhou T, Šimůnek J, Braud I (2021). Adapting HYDRUS-1D to simulate the transport of soil water isotopes with evaporation fractionation. Environ. Model. Softw..

[37] Benettin P (2018). Effects of climatic seasonality on the isotopic composition of evaporating soil waters. Hydrol. Earth Syst. Sci..

[38] Jasechko S, Kirchner JW, Welker JM, McDonnell JJ (2016). Substantial proportion of global streamflow less than three months old. Nat. Geosci..

[39] Ellsworth PZ, Williams DG (2007). Hydrogen isotope fractionation during water uptake by woody xerophytes. Plant Soil.

[40] Groh J (2018). Inverse estimation of soil hydraulic and transport parameters of layered soils from water stable isotope and lysimeter data. Vadose Zonr J..

[41] Dansgaard W (1964). Stable isotopes in precipitation. Tellus A.

[42] Schaap MG, Bouten W, Verstraten JM (1997). Forest floor water content dynamics in a Douglas fir stand. J. Hydrol..

[43] Finkenbiner, C. E. & Good, S. P. HYDRUS Model Data From 2022 Finkenbiner Study On Soil Isotope Separation. *Hydroshare*. 10.4211/hs.a4590c87d0e4454a9a3de60b482d306a (2022).

[44] Daly, C. & McKee, W. A. Meteorological data from benchmark stations at the Andrews Experimental Forest, 1957 to present. *Environmental Data Initiative*. 10.6073/pasta/c021a2ebf1f91adf0ba3b5e53189c84f (2019).

