# Supplementary material for: The extent to which soil hydraulics can explain ecohydrological separation
Source: Nat Commun. 2022 Oct 30;13:6492. doi: 10.1038/s41467-022-34215-7 (PMC9618555; doi:10.1038/s41467-022-34215-7)
Supplement: Supplementary file 1 — Supplementary Information [file 41467_2022_34215_MOESM1_ESM.pdf]

SUPPLEMENTAL INFORMATION

Modeled Parameterization Approach

Soil hydraulic properties for the soil at H.J. Andrews were approximated with the HYDRUS-1D built-in ROSETTA DLL (Dynamically Linked Library) software which implements pedotransfer functions that predict van Genuchten[1] water retention parameters and the saturated hydraulic conductivity. The values used within the pedotransfer function were 27, 35, and 38% sand, silt, and clay, respectively, and a porosity of 60% (these are the same values cited in Brooks et al.[2]). The estimated saturated volumetric water content was 0.55 cm^3/cm^3 and saturated hydraulic conductivity was 65.64 cm/day. The root-mean-square error (RMSE) of the observed and modeled bulk volumetric water content was 0.06 cm^3/cm^3. We used multiple techniques to estimate the soil hydraulic parameters based on observed hydraulic properties at H.J. Andrews and the *VWC* time series from Brooks et al.[2]; this included inverse modeling using the observed soil moisture as a target. Based on RMSE, the ROSETTA DLL outperformed all techniques and was computationally efficient. It is important to note that the objective was not to replicate the observed time series perfectly. No soil evaporation or root water uptake was simulated, as to drive all isotopic differences between soil domains from the parameterized soil physics. All models had an 'Atmospheric' upper boundary condition, and the lower boundary condition was set to 'Free Drainage'.

Modeled Input Precipitation Datasets

We generated precipitation isotope time series that reflected the average and variance of the δ^2H ratio and its correlation with precipitation depth over time. Observed δ^2H in precipitation was sampled in 5 mm increments over the study period[1], but the model required input data in 15-

minute increments. Accordingly, the observed time series was downscaled, using the statistical downscaling method developed by Finkenbiner et al.[3], to correspond with the observed 15-minute precipitation data (H.J. Andrews PRIMNET database[4], Fig. 1a). This statistical downscaling method decomposed the observed precipitation isotope ratio time series into its deterministic and stochastic components. Stochastic components are generated through a Gaussian copula and the deterministic components are fit to the seasonal cycle; both stochastic and deterministic components are generated and combined to produce synthetic realizations of input precipitation isotope ratios. The total input precipitation amount for the 100 days was 101.5 cm. δ^2H ratios were simulated from a Gaussian distribution using the observed mean of -66.7‰, standard deviation of 21.5‰, and Pearson correlation coefficient ρ, ρ(precipitation (cm), δ^2H ratio (‰)), of -0.16. The generated precipitation isotope time series had seasonality effects removed as to drive separations strictly from soil properties or amount effects. Ten unique 15-minute δ^2H time series were generated for each model configuration and each time series captured the statistics of the sampled precipitation (Fig. 1a). We chose to simulate ten time series of δ^2H ratios so as not to tie results to one specific input time series. Additionally, an ensemble size of ten time series was chosen due to the lengthy computational time of each model (~5 min), the amount of time required to process, manipulate, and analyze the output files, and the size of the HYDRUS-1D input/output files (~100 -150 MB).

Modeled Initial Conditions

The soil water's initial δ^2H concentration was unknown and was set equal to the average weighted precipitation. The 100-day time series was repeated three times to remove the effects of the initial condition of the soil's isotopic concentration, and the final 100 days (days 200-300)

were analyzed in the presented results. To explore the effects of changing the initial condition of the isotopic concentration of the soil water, we changed the soil's initial condition to a very high δ^2H value of +200‰. The mobile soil water decreases to average soil water concentrations observed in other model configurations presented in this paper within the 200-day spin up period. Immobile soil water takes much longer, most likely a function of the mass transfer rate between the mobile and immobile domains, and eventually decreases to the average soil water concentrations observed in other model configurations. Therefore, we concluded that changing the initial condition would have minimal impact on the presented results.

Table S1 | The linear regression, p-value, Pearson correlation coefficient, and Spearman correlation coefficient for the isotopic separation between water pore spaces and volumetric water content.

	Linear Regression	p-value	Pearson	Spearman
	**Mobile – Immobile Water v. Volumetric Water Content (cm^3/cm^3)**			
$H_f H_\omega$	y = 3.4x – 1.5	5.00E-03	0.15	0.13
$L_f H_\omega$	y = 6.5x – 2.8	1.69E-08	0.30	0.28
$H_f L_\omega$	y = 3.2x – 1.5	2.00E-02	0.13	0.09
$L_f L_\omega$	y = 6.6x – 2.9	3.60E-08	0.30	0.27
	**Drainage – Mobile Water v. Volumetric Water Content (cm^3/cm^3)**			
0_f	y = -7.6x + 3.5	2.06E-06	-0.26	-0.23
$H_f H_\omega$	y = 12.2x – 5.4	1.61E-12	0.38	0.37
$L_f H_\omega$	y = 2.2x – 0.9	1.90E-01	0.07	0.11
$H_f L_\omega$	y = 15.2x – 6.6	7.97E-15	0.41	0.42
$L_f L_\omega$	y = 2.7x – 1.1	1.20E-01	0.09	0.12
	**Drainage – Immobile Water v. Volumetric Water Content (cm^3/cm^3)**			
$H_f H_\omega$	y = 15.6x – 6.9	3.60E-20	0.48	0.47
$L_f H_\omega$	y = 8.7x – 3.7	1.25E-09	0.33	0.35
$H_f L_\omega$	y = 18.4x – 8.1	5.53E-22	0.50	0.50
$L_f L_\omega$	y = 9.3x – 4.0	9.21E-10	0.33	0.35

Table S2 | The linear regression, p-value, Pearson correlation coefficient, and Spearman correlation coefficient for the isotopic separation between soil pore spaces at the end of a day and daily accumulated precipitation.

	Linear Regression	p-value	Pearson	Spearman
	**Mobile – Immobile Water v. Precipitation (cm)**			
H_fH_ω	y = -0.001x – 0.1	8.89E-01	-0.03	-0.21
L_fH_ω	y = 0.01x – 0.2	4.03E-01	0.15	0.33
H_fL_ω	y = -0.002 – 0.2	7.75E-01	-0.05	0.17
L_fL_ω	y = 0.004 – 0.3	4.32E-01	0.14	0.33
	**Drainage – Mobile Water v. Precipitation (cm)**			
0_f	y = -0.01x + 0.5	5.25E-01	-0.12	-0.34
H_fH_ω	y = 0.02x – 0.8	1.48E-02	0.42	0.36
L_fH_ω	y = 0.01x – 0.2	1.22E-01	0.28	0.06
H_fL_ω	y = 0.02x – 0.8	1.48E-02	0.42	0.43
L_fL_ω	y = 0.01x – 0.2	1.23E-01	0.27	0.07
	**Drainage – Immobile Water v. Precipitation (cm)**			
H_fH_ω	y = 0.02x – 0.9	2.00E-02	0.40	0.54
L_fH_ω	y = 0.02x – 0.4	1.00E-02	0.44	0.37
H_fL_ω	y = 0.02x – 1.0	2.00E-02	0.40	0.54
L_fL_ω	y = 0.02x – 0.4	1.40E-02	0.42	0.37

Table S3 | The linear regression, p-value, Pearson correlation coefficient, and Spearman correlation coefficient for the isotopic separation between soil pore spaces and drainage.

	Linear Regression	p-value	Pearson	Spearman
	**Mobile – Immobile Water v. Drainage (cm)**			
$H_f H_\omega$	y = 0.1x – 0.2	2.20E-02	0.13	0.18
$L_f H_\omega$	y = 0.2x – 0.4	3.54E-05	0.23	0.34
$H_f L_\omega$	y = 0.2x – 0.4	6.00E-02	0.10	0.14
$L_f L_\omega$	y = 0.1x – 0.3	3.19E-05	0.23	0.33
	**Drainage – Mobile Water v. Drainage (cm)**			
0_f	y = -0.2x + 0.7	2.50E-04	-0.20	-0.25
$H_f H_\omega$	y = 0.4x -1.0	5.21E-12	0.37	0.40
$L_f H_\omega$	y = 0.1x – 0.1	5.70E-02	0.06	0.09
$H_f L_\omega$	y = 0.5x -1.1	2.25E-12	0.37	0.39
$L_f L_\omega$	y = 0.1x – 0.1	3.05E-01	0.57	0.11
	**Drainage – Immobile Water v. Drainage (cm)**			
$H_f H_\omega$	y = 0.5x – 1.2	7.41E-18	0.45	0.56
$L_f H_\omega$	y = 0.2x – 0.4	6.73E-06	0.25	0.36
$H_f L_\omega$	y = 0.6x – 1.4	1.83E-17	0.45	0.52
$L_f L_\omega$	y = 0.2x – 0.5	9.35E-06	0.24	0.37

Table S4 | Summary of all 650 model parameter configurations explored in the study.

Description	Units	Parameter Range
Volumetric water content	cm^3/cm^3	0.35 – 0.55
Saturated hydraulic conductivity	cm/day	40 – 80
Increasing or decreasing the total input precipitation (=101.5 cm)	As a percent of accumulated precipitation (%)	40 – 120
Pearson correlation coefficient (ρ) between precipitation amount (P) and its δ^2H concentration	Unitless	-0.4 – 0.0

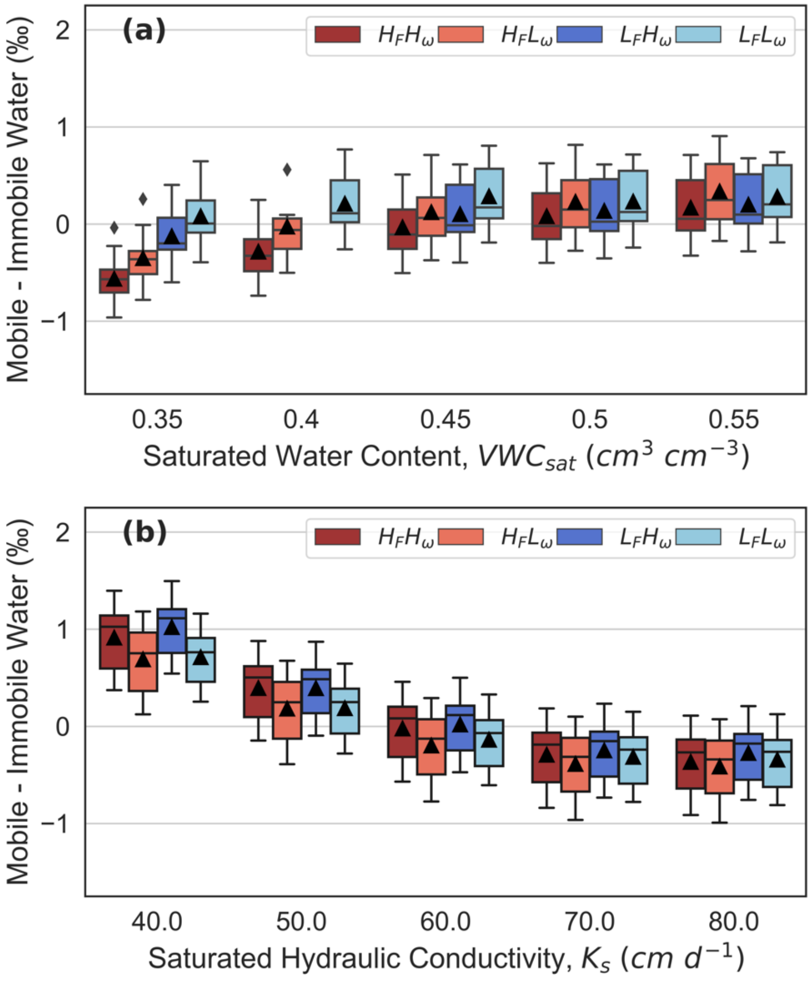

Figure S1 | The influence of modeled soil hydraulic parameters on the average δ^2H difference between mobile and immobile soil water. The influence of varying **a** soil saturated water content and **b** soil saturated hydraulic conductivity on the average δ^2H difference between drainage mobile or immobile soil water. Differences are show as boxplots for models with high and low immobile fractions (H_f and L_f) and transfer rates (H_ω and L_ω) as well as for a single porosity column (0_f). Each boxplot represents flux and volumetrically weighted averaged differences, with the box spanning upper and lower quartiles, whiskers extending 1.5 times the interquartile range, diamonds as outliers, and black triangles indicating the mean calculated from 10 simulations.

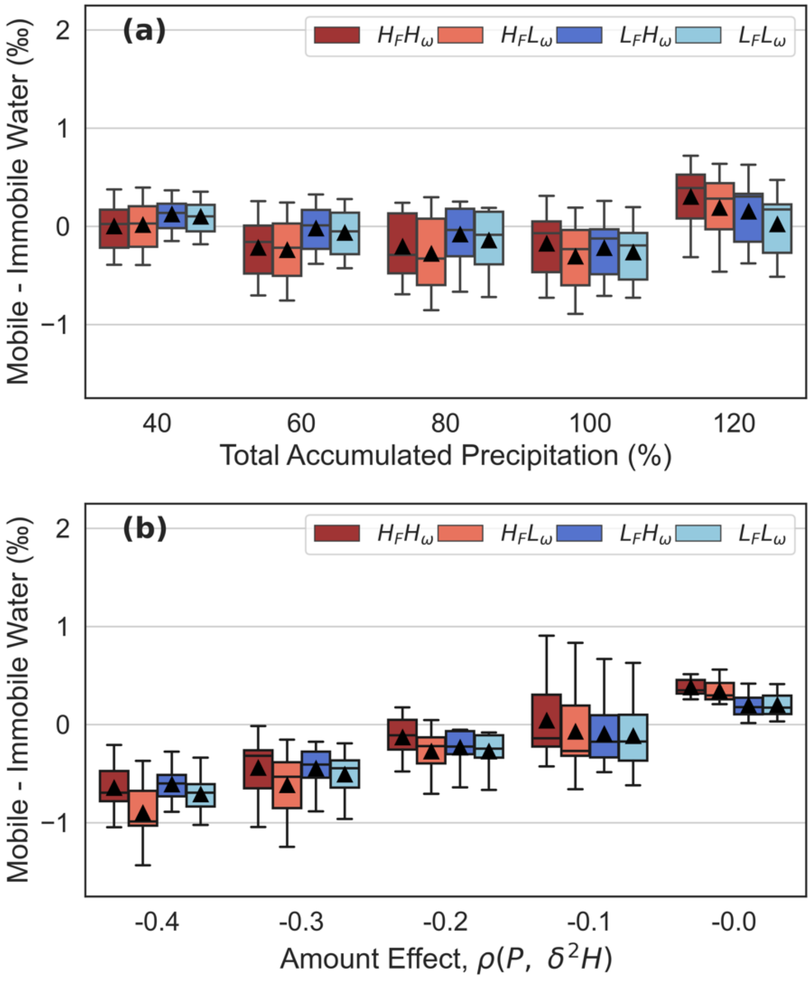

Figure S2 | The influence of modeled climate parameters on average δ^2H difference between mobile and immobile soil water. The influence of **a** increasing or decreasing the total input precipitation and **b** varying the amount effect's negative correlation on the average δ^2H difference between mobile and immobile soil water. Differences are show as boxplots for models with high and low immobile fractions (H_f and L_f) and transfer rates (H_ω and L_ω) as well as for a single porosity column (0_f). Each boxplot represents flux and volumetrically weighted averaged differences, with the box spanning upper and lower quartiles, whiskers extending 1.5 times the interquartile range, diamonds as outliers, and black triangles indicating the mean calculated from 10 simulations.

SUPPLEMENTARY REFERENCES

1. van Genuchten, M.T. A closed-form equation for predicting the hydraulic conductivity of unsaturated soils. *Soil Science Society of America Journal* 44, 892-898 (1980). https://doi.org/10.2136/sssaj1980.03615995004400050002x

2. Brooks, J. R., Barnard, H., Coulombe, R. et al. Ecohydrologic separation of water between trees and streams in a Mediterranean climate. *Nature Geosci* 3, 100–104 (2010). https://doi.org/10.1038/ngeo722

3. Finkenbiner, C.E., Good, S.P., Allen, S.T., Fiorella, R.P., and Bowen, G.J. A statistical method for generating temporally downscaled geochemical tracers in precipitation. *J Hydrometerol* 22, 1473-1486 (2021). https://doi.org/10.1175/JHM-D-20-0142.1

4. Daly, C. and McKee, W.A. Meteorological data from benchmark stations at the Andrews Experimental Forest, 1957 to present ver 36. Environmental Data Initiative (2019). https://doi.org/10.6073/pasta/c021a2ebf1f91adf0ba3b5e53189c84f (Accessed 2021-04-21).
